# Predicting the Lifespan of Twisted String Actuators Using Empirical and Hybrid Machine Learning Approaches

**DOI:** 10.3390/s25237387

**Published:** 2025-12-04

**Authors:** Hai Nguyen, Chanthol Eang, Seungjae Lee

**Affiliations:** Department of Computer Science and Engineering/Intelligent Robot Research Institute, Sun Moon University, Asan 31460, Republic of Korea; hainguyen091520@gmail.com (H.N.); ngchanthol1@gmail.com (C.E.)

**Keywords:** twisted string actuator (TSA), fatigue life prediction, physics-guided machine learning, hybrid XGBoost, reliability modeling, uncertainty quantification, predictive maintenance

## Abstract

Predicting the fatigue lifespan of Twisted String Actuators (TSAs) is essential for improving the reliability of robotic and mechanical systems that rely on flexible transmission mechanisms. Traditional empirical approaches based on regression or Weibull distribution analysis have provided useful approximations, yet they often struggle to capture nonlinear dependencies and stochastic influences inherent to real-world fatigue behavior. This study introduces and compares four machine learning (ML) models—Linear Regression, Random Forest, XGBoost, and Gaussian Process Regression (GPR)—for predicting TSA lifespan under varying weight (W), number of strings (N), and diameter (D) conditions. Building upon this comparison, a hybrid physics-guided model is proposed by integrating an empirical fatigue life equation with an XGBoost residual-correction model. Experimental data collected from repetitive actuation tests (144 valid samples) served as the basis for training and validation. The hybrid model achieved an R^2^ = 0.9856, RMSE = 5299.47 cycles, and MAE = 3329.67 cycles, outperforming standalone ML models in cross-validation consistency (CV R^2^ = 0.9752). The results demonstrate that physics-informed learning yields superior interpretability and generalization even in limited-data regimes. These findings highlight the potential of hybrid empirical–ML modeling for component life prediction in robotic actuation systems, where experimental fatigue data are scarce and operating conditions vary.

## 1. Introduction

Twisted String Actuators (TSAs) have emerged as one of the most promising solutions for compact, lightweight, and high-force robotic actuation. By converting rotational motion into linear displacement through the twisting of flexible strings or cables, TSAs achieve remarkable mechanical efficiency, generating high output forces relative to their small mass and volume [1,2]. This unique principle enables the design of efficient transmission systems with advantages such as compactness, mechanical simplicity, energy-efficient, and high force-to-weight ratio, making them ideal for wearable robotics, assistive devices, and soft actuation systems [3,4].

Recent research highlights the versatility of TSAs across diverse engineering domains. For instance, adjustable-offset TSA mechanisms have been developed to provide variable transmission ratios and precise motion control [3], while haptic force-rendering TSAs enable realistic feedback in immersive human–machine interfaces [4]. Similarly, fabric-integrated wearable gloves employing TSA actuation have shown potential in rehabilitation and manual handling tasks, offering compactness without compromising strength [5,6]. At larger scales, the NASA Ames Research Center explored impedance-controlled TSA architectures for tensegrity robots, where lightweight, compliant actuation systems are essential for safe planetary exploration and adaptive locomotion [7]. More recently, Zheng et al. [8] applied a fully data-driven modeling strategy to twisted string mechanisms for anthropomorphic robotic hands, demonstrating that nonlinear actuator behaviors can be captured through hybrid AI-based representations while maintaining mechanical compliance and control precision. These developments illustrate the growing convergence between physical actuation design and intelligent modeling paradigms.

Despite these advances, the fatigue life and durability of TSAs remain a major limitation for long-term deployment. Each twist–untwist cycle induces torsional and bending stresses that gradually degrade the wire material, eventually leading to micro-fracture and failure. The degradation rate is influenced by multiple interdependent factors, including applied load, string diameter, number of wires, material properties, and frictional losses, as well as environmental conditions such as temperature and humidity. These nonlinear interactions render lifespan prediction a highly complex problem, yet accurate prediction is crucial for ensuring reliability, safety, and predictive maintenance in robotic and assistive systems.

Historically, empirical and statistical life prediction models, such as those based on Weibull distributions or regression analysis, have been employed to model mechanical fatigue. These methods offer clear interpretability and strong physical grounding but often rely on simplifying assumptions, such as linearity or independence among variables. While effective for isolated parameters, such formulations cannot fully capture the nonlinear coupling effects observed in twisted string mechanisms, where geometric deformation, internal friction, and microstructural wear interact dynamically over time. Consequently, empirical models tend to underperform when extrapolated beyond calibrated experimental conditions.

In recent years, machine learning (ML) has gained attention as a powerful tool for nonlinear regression and multivariate modeling in mechanical systems. Algorithms such as Random Forest (RF), Extreme Gradient Boosting (XGBoost), and Gaussian Process Regression (GPR) can learn complex mappings between input parameters and observed fatigue outcomes, even from small datasets. However, purely data-driven methods are often criticized as “black-box” models, offering high predictive accuracy at the expense of physical interpretability. For safety-critical or high-reliability applications, such as TSA-driven exoskeletons and space robotics, this lack of interpretability poses a significant barrier to practical adoption. Recent industrial studies have also demonstrated the promise of interpretable hybrid AI frameworks for reliability estimation and equipment lifespan prediction [9], reinforcing the need for models that combine transparency with predictive strength.

To address these challenges, this study proposes a hybrid physics-guided machine learning framework that integrates empirical knowledge with data-driven learning. The approach combines a Weibull-based empirical model, which encodes deterministic physical relationships with an XGBoost residual learner, which models unaccounted nonlinear deviations. This Hybrid Empirical–XGBoost model aims to achieve three primary goals:

1. Enhance predictive accuracy by learning residual deviations beyond empirical approximations;
2. Maintain physical interpretability through embedded physics-based priors;
3. Improve generalization in small-sample experimental contexts typical of actuator testing.

The framework is evaluated using a dataset of 144 TSA fatigue life samples under varied load and geometry conditions. Five modeling paradigms are systematically compared: (i) Linear Regression (LR), (ii) Random Forest (RF), (iii) XGBoost (XGB), (iv) Gaussian Process Regression (GPR), and (v) the proposed hybrid empirical–XGBoost model.

The major contributions of this study are summarized as follows:

- Dataset Development: Establishment of a consolidated experimental dataset for TSA fatigue life prediction covering diverse loading and geometric conditions.
- Modeling Framework: Development and comparative evaluation of five predictive approaches, including a hybrid physics-guided architecture combining empirical and ML components.
- Interpretability and Practical Deployment: Implementation of a Python-based graphical user interface (GUI) enabling real-time prediction, visualization, and parameter sensitivity analysis for engineering applications.

## 2. Related Works

Understanding the fatigue behavior and lifespan of Twisted String Actuators (TSAs) requires integrating insights from multiple research domains, ranging from traditional empirical fatigue modeling to data-driven machine learning (ML) prediction, and hybrid physics-informed modeling frameworks.

This section reviews prior literature in these areas, with emphasis on methodologies that have influenced the development of our hybrid empirical–machine learning approach, which includes exploring traditional empirical and statistical models of fatigue life, especially Weibull-based reliability analysis; expanding on Linear Regression (LR) and interpretable modeling techniques used in mechanical reliability. Then, we will cover ensemble-based ML models, namely Random Forest (RF) and Extreme Gradient Boosting (XGBoost), followed by discussing Gaussian Process Regression (GPR) as a Bayesian framework for uncertainty quantification. Finally, we review physics-informed and hybrid approaches that unify empirical modeling with modern ML algorithms, providing the theoretical foundation for our hybrid TSA lifespan predictor.

### 2.1. Empirical and Statistical Approaches to Mechanical Fatigue

Empirical reliability analysis has long been the foundation of fatigue life prediction for mechanical systems. Conventional regression models approximate deterministic relationships between operating load, cycle count, and geometry but generally rely on simplified functional assumptions, linear or power–law relations, that fail under complex, nonlinear fatigue mechanisms.

To capture the stochastic nature of material failure, the Weibull distribution became the dominant probabilistic tool. It describes time-to-failure as a statistical process defined by shape and scale parameters that quantify the likelihood and spread of failure events. Jesús et al. [10] validated the Weibull distribution for mechanical fatigue modeling, demonstrating its flexibility in characterizing lifetime dispersion. Fakoor et al. [11] introduced parameter modifications to improve predictive precision, and Wang et al. [12] extended the technique to mechanical-part fatigue analysis. Kundu et al. [13] applied a Weibull-based accelerated-life regression for remaining useful life (RUL) prediction under multiple loads, while Herzog [14] merged Weibull reliability metrics with neural networks for bearing-life estimation. Mohammad et al. [15] linked Weibull distributions with acoustic-emission features to forecast fatigue in SAE-1045 steel.

Although Weibull regression excels at handling randomness and providing interpretability, it is constrained by its parametric simplicity. In TSA systems, where lifespan depends jointly on weight (W), diameter (D), and number of strings (N), traditional models often fail to describe nonlinear cross-effects or manufacturing variability. Consequently, modern studies increasingly adopt machine learning techniques capable of capturing these higher-order dependencies directly from data.

### 2.2. Linear Regression and Interpretable Modeling

Before the rise of nonlinear ensemble algorithms, Linear Regression (LR) was the primary modeling tool for mechanical reliability and predictive maintenance tasks. Its enduring appeal lies in its simplicity, transparency, and strong interpretability, making it ideal for understanding how individual variables affect system lifespan.

Recent works continue to highlight the importance of LR in engineering diagnostics. Yıldırım et al. [16] demonstrated LR’s reliability in NASA C-MAPSS datasets for predictive maintenance, establishing its effectiveness in baseline life estimation for turbine systems. Lajos Höfler [17] assessed LR’s regression accuracy using sensor data in chemical systems, showing that linear models can yield strong predictive performance when features are physically meaningful. Kruschel et al. [18] challenged the traditional notion that interpretability must trade off against accuracy, presenting linear and generalized additive models that achieved near–black-box performance in structured data environments. Similarly, Long et al. [19] emphasized the synergy between interpretable ML and sensor fusion for real-time industrial health monitoring.

Beyond interpretability, LR serves as a benchmark for evaluating nonlinear learners such as Random Forest (RF) and XGBoost. Christos et al. [20] used polynomial and Linear Regression for wind-turbine energy prediction, and Schaeffer et al. [21] analyzed high-dimensional LR stability. Additionally, Schauer et al. [22] introduced hardware-efficient implementations of interpretable ML models for sensor-based diagnostics, improving the practicality of regression-based reliability prediction.

In TSA lifespan prediction, LR provides a critical baseline. While it cannot represent nonlinear degradation mechanisms or material fatigue saturation, it enables feature-level analysis, identifying which design variables (e.g., load or diameter) most influence actuator endurance. This interpretability makes it invaluable for model validation and physical understanding prior to adopting more complex ML frameworks.

### 2.3. Ensemble Learning: Random Forest and XGBoost

Ensemble methods such as Random Forest (RF) and Extreme Gradient Boosting (XGBoost) have revolutionized data-driven fatigue modeling by combining interpretability with strong nonlinear representation.

RF constructs an ensemble of decision trees trained on random feature subsets, mitigating overfitting and enabling robust feature importance analysis. Fatima et al. [23] and Imani et al. [24] demonstrated RF’s efficiency across mechanical prediction tasks, while Suenaga et al. [25] validated its superiority over shallow neural networks for structural performance estimation. Wohlwend [26] and Hamidou et al. [27] highlighted RF’s consistent accuracy in fault diagnosis and anomaly detection. Yang et al. [28] applied RF and XGBoost to machine-failure prediction, confirming their generalization ability under imbalanced datasets.

Building upon RF, XGBoost employs iterative gradient boosting to minimize residual errors with strong regularization. The primary distinction between the two methods lies in how decision trees are aggregated. RF builds trees in parallel, while XGBoost constructs them sequentially to optimize performance [29]. This distinction allows XGBoost to achieve higher predictive precision, particularly for small and noisy datasets such as TSA fatigue experiments.

XGBoost extends this paradigm through boosted gradient optimization, sequentially reducing residual errors to achieve high precision even on small datasets, like the 144-sample TSA dataset used in this study. Its built-in regularization (L1/L2) prevents overfitting, while its scalability enables fast convergence. RF and XGBoost together represent a balance between explainability, efficiency, and predictive accuracy, making them ideal candidates for small-scale experimental fatigue data.

### 2.4. Gaussian Process Regression and Uncertainty Quantification

Gaussian Process Regression (GPR) provides a probabilistic framework for nonlinear modeling, explicitly representing both predicted means and uncertainties. Unlike deterministic learners, GPR outputs confidence intervals, an essential feature for safety-critical mechanical systems, where risk-aware decision-making is vital.

Li et al. [30] introduced GPR as an intuitive framework for modeling both predictions and associated confidence intervals, while Li et al. [31] implemented deep Bayesian GPR for medical uncertainty estimation. Manfredi [32] and Bilionis et al. [33] developed hybrid polynomial chaos–GPR models to enhance Bayesian sensitivity analysis, enabling uncertainty propagation in mechanical systems. Oakley [34] provided a foundational overview of GPR for engineers, illustrating its practical utility in uncertainty-aware regression.

In TSA fatigue modeling, where datasets are limited, GPR’s nonparametric nature makes it highly suitable for capturing smooth nonlinear relationships and generating credible uncertainty intervals. This capability enhances trust in predictions, essential for mission-critical actuators or robotic systems.

### 2.5. Physics-Informed and Hybrid Modeling Frameworks

While pure data-driven ML models often achieve high accuracy, they lack physical interpretability, hindering their acceptance in engineering applications. Conversely, empirical models are interpretable but limited in adaptability. To reconcile these paradigms, recent studies have proposed hybrid and physics-informed learning frameworks that integrate domain knowledge into ML architectures.

Raissi et al. [35] pioneered the Physics-Informed Neural Network (PINN), integrating differential equations directly into the training process. Farea et al. [36] and Zhang et al. [37] advanced this concept by embedding physical constraints into neural architectures. SoftServe [38] emphasized the growing industrial relevance of PINNs, describing their role in merging AI and engineering physics, while Luo et al. [39] reviewed its broad applicability across engineering PDE problems. Wesselkamp et al. [40] extended these ideas into Process-Informed Neural Networks (PrINNs), demonstrating improved inference under scientific consistency.

Beyond PINNs, hybrid empirical–ML regression models have gained traction in mechanical reliability and fatigue life prediction. In these frameworks, an analytical or empirical model provides a physics-consistent baseline, while an ML model learns the residual deviations caused by nonlinear effects, manufacturing variability, or microstructural changes. This approach retains interpretability while improving adaptability to complex real-world patterns.

The present study builds on this emerging paradigm by designing a hybrid framework tailored specifically to Twisted String Actuators. Unlike generic hybrid models, our approach integrates a TSA-specific empirical fatigue life equation derived from torsional and geometric mechanics and constrains the machine learning component (XGBoost) to operate on physically meaningful variables such as weight, diameter, and wire configuration. This targeted integration enables the model to capture global degradation trends through the empirical component while allowing XGBoost to represent localized nonlinear effects that empirical models alone cannot describe.

Despite significant advancements across empirical, interpretable, ensemble, and hybrid paradigms, predicting TSA fatigue life remains challenging due to limited datasets, nonlinear parameter coupling, and material variability. Conventional Weibull or linear models cannot capture these cross-effects, while purely ML-based methods often lose physical meaning. The need, therefore, is for a lightweight hybrid model that leverages empirical domain knowledge and the nonlinear flexibility of ML, precisely the direction taken in this study.

## 3. Methodology

This section describes the dataset structure, preprocessing steps, modeling procedures, and evaluation protocols used to predict the fatigue lifespan of Twisted String Actuators (TSAs). The methodological workflow integrates empirical modeling, machine learning (ML) algorithms, and a hybrid physics-guided approach, culminating in an interactive GUI for model testing and validation.

**Figure 1:**
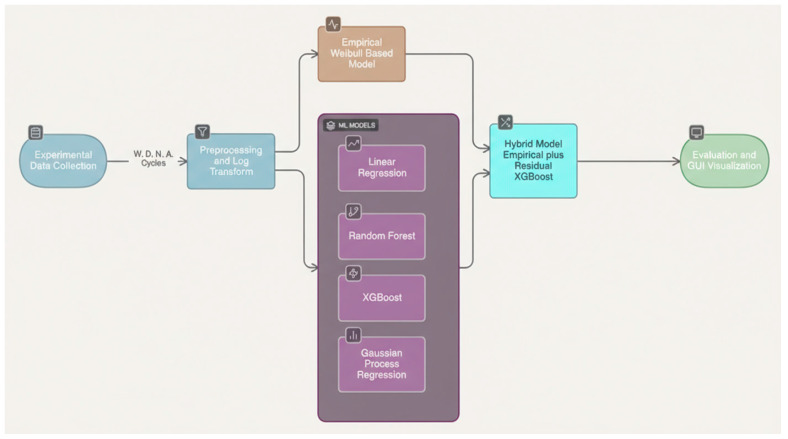
Methodology workflow.

Figure 1 illustrates a schematic overview of the full modeling pipeline used in this study. The process begins with experimental data collection of five key parameters related the wires, which are weight (W), diameter (D), number of wires (N), cross-sectional area (A) and lifespan, or number of execution (cycles) until the wire break, followed by preprocessing and log transformation for variance stabilization. The empirical Weibull-based model establishes a deterministic baseline, while four machine learning models (Linear Regression, Random Forest, XGBoost, and Gaussian Process Regression) provide nonlinear, data-driven predictions. The hybrid Empirical + Residual XGBoost model integrates both physics and data learning. Final evaluation and visualization are performed through a Python-based GUI.

### 3.1. Dataset and Experimental Setup

#### 3.1.1. Experimental Design

Twisted String Actuators (TSAs) were subjected to cyclic twisting–untwisting operations under controlled laboratory conditions until complete wire fracture occurred. Each actuator specimen consisted of multiple steel or nylon wires connected to a rotary motor applying constant tension through a load cell. The cycles to failure were recorded automatically through a digital counter linked to the actuator control system. Environmental conditions, such as temperature (~25 °C), humidity (~50%), and rotational speed (150 rpm), were maintained constant to minimize uncontrolled variance.

All twisted string specimens were fabricated using LIROS D-Pro SK78, which originates from LIROS Ropes/Karlskron, Bavaria, Germany, a high-performance rope constructed from 100% Dyneema^®^ SK78 fibers. This material is manufactured under strict industrial quality-control processes, including a proprietary heat-stretch stabilization procedure that ensures consistent breaking strength, elongation characteristics, density, and surface finish across production batches. To minimize material variability, all samples were sourced from the same manufacturing lot (or adjacent batch) and were subjected to visual inspection and basic pre-tension checks before testing. Although minor microstructural differences may remain, the standardized nature of SK78 rope and these pre-test screening procedures justify the assumption of material uniformity for the purpose of fatigue life modeling.

Each experimental configuration corresponded to a unique combination of applied weight (W), string diameter (D), and number of wires (N). The resulting fatigue life (C) values were measured in cycles until mechanical failure, reflecting the actuator’s endurance under repetitive mechanical stress. A total of 144 valid experimental observations were retained after removing incomplete or corrupted entries, representing a balanced mix of load and geometry variations. This dataset forms the basis for both empirical regression and machine learning model training.

Although the dataset consists of 144 fatigue life samples, this scale is typical for Twisted String Actuator testing, where each experiment requires thousands of cycles and ends in destructive failure. Consequently, data collection is inherently slow, resource-intensive, and physically constrained. To mitigate risks associated with small datasets, the modeling pipeline incorporates repeated 5-fold cross-validation, nested hyperparameter tuning, and a hybrid regression structure in which the empirical component captures global degradation trends while the machine learning component learns only localized nonlinear residuals. These measures collectively reduce variance and improve robustness despite the limited sample size.

**Figure 2:**
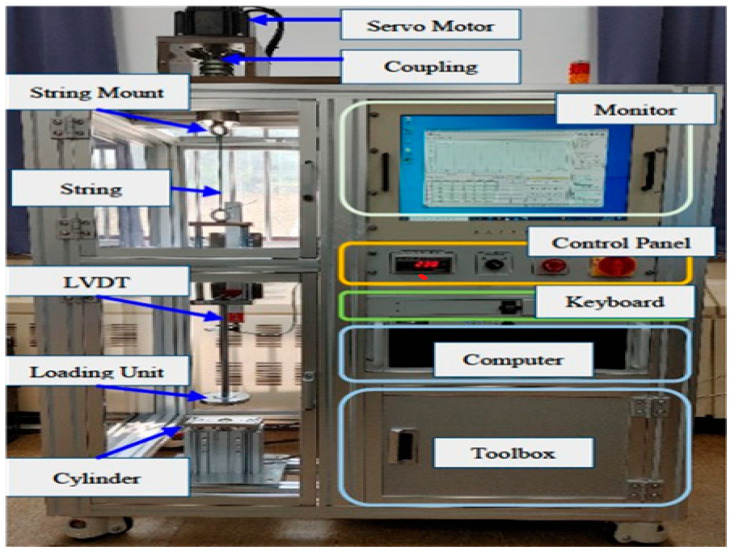
Experimental setup for Twisted String Actuator (TSA) fatigue testing.

The experimental setup used for fatigue testing of the Twisted String Actuators is shown in Figure 2. The test bench consists of a servo-motor–driven rotary system connected to a string-mounting frame, a load unit with a cylinder for tension application, and a displacement sensor (LVDT) for real-time elongation tracking. The entire assembly integrates a control panel, data-acquisition monitor, and computer system for automatic recording of cycles to failure, ensuring reproducible test conditions. This apparatus enabled precise control of loading parameters (W, D, N) and accurate detection of fracture points across all 144 experimental runs.

#### 3.1.2. Variables

- Input Variables:
  ◦ Weight (W): 15–25 kg applied tension.
  ◦ Diameter (D): 1.0–2.0 mm string thickness.
  ◦ Number of Strings (N): 2–7 parallel wires.
  ◦ Cross-Sectional Area (A): computed as $A=\pi (D/2{)}^{2}$
- Output Variable:
  ◦ Cycles to Failure (C): total number of actuation cycles until wire fracture.

All of variables are summarized in Table 1.

#### 3.1.3. Data Acquisition and Organization

Raw measurements were recorded in structured CSV blocks corresponding to three load categories (15, 20, 25 kg). Within each load block, multiple-diameter string count combinations were tested to capture multivariate behavior. Data integrity was verified through duplicate checks and physical inspection logs. The dataset was programmatically parsed using Python (pandas) into a unified data frame for subsequent preprocessing and analysis.

#### 3.1.4. Data Preprocessing

To ensure statistical consistency and model convergence, preprocessing steps were applied as follows:

1. Cleaning: Invalid entries such as zero-cycle measurements, corrupted logs, or nonsensical values were removed. These errors originated from counter resets, missing LVDT frames, or automatic safety stops and did not represent meaningful physical noise. Normal experimental noise from sensors was preserved. NaN values primarily resulted from intermittent sensor malfunctions, including incomplete cycle-count logs and temporary LVDT readout interruptions.
2. Feature Engineering: The cross-sectional area was computed and added as a predictor.
3. Normalization: Input features were standardized to zero mean and unit variance.
4. Transformation: The output variable (Cycles to Failure) was log-transformed (log(1+y)) to stabilize variance and reduce heteroscedasticity.
5. Train–Test Split: An 80/20 split ensured balanced representation of parameter ranges across training and validation sets.

This workflow was implemented in Python 3.13 using NumPy, pandas, and scikit-learn, allowing reproducible data handling and model evaluation.

### 3.2. Empirical Baseline Model

Prior to machine learning training, an empirical life prediction equation was used as the baseline. The model was derived from Weibull distribution-based life analysis and multiple regression fitting, capturing the influence of weight, number of strings, and diameter on lifespan. The formulation captures the primary physical dependencies between actuator geometry and applied load:

(1) $$C_{emp}=a\times D^{b}\times N^{c}\times W^{-d},$$

where

- $C_{\mathrm{emp}}$ are the predicted cycles to failure,
- D is the string diameter,
- N is the number of parallel wires,
- W is the applied weight, and
- a, b, c, d are regression coefficients estimated via least-squares fitting on the log-transformed data.

This empirical approach provides a deterministic interpretation of fatigue behavior: lifespan increases with diameter and string count but decreases with applied load. Although highly interpretable, the model cannot fully represent nonlinear interactions or manufacturing variability. The empirical model serves two key purposes:

1. Benchmarking: Provides a deterministic reference for evaluating the gain achieved by ML models.
2. Hybrid Prior: Acts as a physics-based prior in the hybrid model, constraining learning to physically meaningful domains.

### 3.3. Machine Learning Models

Four machine learning algorithms were implemented and benchmarked against the empirical model: Linear Regression (LR), Random Forest (RF), Extreme Gradient Boosting (XGBoost), and Gaussian Process Regression (GPR). All models used identical training and validation splits to ensure comparability. The entire workflow was executed in Python using scikit-learn and XGBoost packages.

The LR model assumes additive linear relationships and serves as a transparent statistical benchmark. It estimates a weighted combination of features to predict fatigue life, enabling straightforward physical interpretation of coefficients. While limited in flexibility, LR is essential for verifying the directionality and proportional influence of each factor, ensuring dataset consistency before applying more complex learners.

RF constructs an ensemble of decorrelated decision trees using bootstrap aggregation, effectively capturing nonlinear interactions. Each tree contributes to the ensemble’s final output via averaging, which mitigates variance and enhances robustness. Feature importance derived from Gini impurity consistently identified diameter and number of strings as dominant predictors, followed by cross-sectional area and load. However, the model displayed mild overfitting due to the limited dataset, prompting the inclusion of gradient-based regularized methods.

XGBoost iteratively constructs trees that correct the residuals of previous ones, using gradient optimization and built-in regularization (L1/L2 penalties). This allows precise modeling of nonlinear dependencies between load, geometry, and fatigue lifespan. Optimal hyperparameters determined via GridSearchCV were n_estimators = 300, learning_rate = 0.05, max_depth = 4, subsample = 0.9, colsample_bytree = 0.9. This configuration yielded stable convergence with limited data, achieving high R^2^ scores and low error variance under 5-fold cross-validation.

The GPR model provides a probabilistic approach for both prediction and uncertainty quantification. Using a composite kernel comprising a Radial Basis Function (RBF) and WhiteKernel, it models smooth nonlinear trends while accounting for observation noise. Beyond accuracy, GPR yields confidence intervals for each prediction, an essential property for reliability-critical applications such as robotic actuation and aerospace mechanisms.

All models were trained using the log-transformed target and evaluated on the test set via R^2^, RMSE, and MAE metrics. Cross-validation (5-fold) was applied to measure stability and generalization.

### 3.4. Hybrid Physics-Guided XGBoost Model

#### 3.4.1. Hybrid Design Philosophy

To combine the interpretability of physics-based modeling with the flexibility of data-driven learning, a hybrid model was developed that embeds the empirical fatigue life equation within a residual learning structure. The hybrid approach adheres to the principle of Physics-Guided Machine Learning (PGML), constraining data-driven optimization through deterministic scientific laws to preserve physical plausibility.

The proposed hybrid framework combines the physics-based empirical model with a data-driven residual learner using XGBoost. The approach follows a two-stage process:

1. Stage 1—Empirical Prediction: the deterministic model computes the baseline lifespan ($C_{emp}$) for each sample based on known physical relationships between L, D, and N.
2. Stage 2—Residual Learning: an XGBoost regressor is trained to predict the residual error ($\Delta C=C_{obs}-C_{emp}$), capturing systematic nonlinearities unexplained by the baseline equation.

The final prediction is thus computed as follows:

(2) $$C_{hybrid}=C_{emp}+f_{XGB}(W,D,N,A)$$

where $f_{XGB}$ represents the learned correction function.

This additive correction structure ensures that the model respects empirical monotonic trends (e.g., lifespan decreases with increasing load) while capturing subtle nonlinear deviations due to microstructural or tribological effects.

Unlike previously reported hybrid frameworks that simply combine an analytical model with a residual learning regressor, the hybrid design used in this study incorporates several TSA-specific innovations. The empirical component is derived directly from the torsional deformation mechanics of twisted string transmission, enabling it to capture the global trend of fatigue degradation that arises from geometric shortening and load-dependent friction. The XGBoost component is intentionally constrained to learn only the localized nonlinear deviations associated with micro-slippage, strand compaction, surface friction evolution, and material hysteresis, which are behaviors that are highly characteristic of TSA actuation but not modeled in conventional fatigue equations. By structuring the hybrid model such that the empirical term governs the physically consistent prediction trend while XGBoost refines only the complex residual patterns, the proposed method ensures both interpretability and enhanced predictive accuracy. This TSA-specific formulation differentiates our approach from existing additive hybrid frameworks and provides a tailored solution for accurately modeling twisted string fatigue behavior.

#### 3.4.2. Implementation and Advantages

The hybrid model was implemented in Python (scikit-learn + XGBoost) and serialized as a portable .pkl file for real-time deployment.

Advantages include the following:

- Higher Predictive Fidelity: Learns nonlinear effects beyond analytical limits.
- Physical Coherence: Retains deterministic relationships to prevent unphysical extrapolation.
- Data Efficiency: Achieves strong accuracy with small datasets due to empirical priors.
- Interpretability: Enables feature importance analysis to identify dominant fatigue drivers.

This structure mirrors modern trends in physics-informed AI, bridging the gap between classical reliability theory and adaptive learning systems.

### 3.5. Graphical User Interface (GUI) Implementation

A Tkinter-based GUI was developed to enable real-time testing and visualization. It serves as both an educational and practical interface for engineers to interact with the trained models. The interface allows users to perform the following:

- Input experimental or hypothetical actuator parameters (W, D, N, A).
- Instantly compute fatigue life predictions from all five models (Empirical, LR, RF, XGB, GPR, Hybrid).
- Visualize comparative outputs, feature importances, and predicted lifespans.
- Export results for design optimization or validation reports.

Internally, the GUI loads serialized model files and executes predictions concurrently, ensuring consistency with Python-based results. This tool not only provides an accessible visualization platform for research validation but also serves as a design support interface for actuator development and educational demonstrations in robotic systems engineering.

### 3.6. Evaluation Metrics

Model performance was quantified using four complementary statistical metrics: the coefficient of determination (R^2^), root mean square error (RMSE), mean absolute error (MAE), and cross-validated R^2^ (CV R^2^).

- Coefficient of Determination (R^2^): R^2^ measures how much of the variance in the observed lifespan values is explained by the model. It is defined as

(3) $$R^{2}=1-\frac{\sum_{i}(y_{i}-{\hat{y}}_{i}{)}^{2}}{\sum_{i}(y_{i}-\overset{ˉ}{y}{)}^{2}}$$

where $y_{i}$ and ${\hat{y}}_{i}$ denote actual and predicted values, and $\overset{ˉ}{y}$ is the sample mean. An $R^{2}$ close to 1 indicates strong explanatory power; a negative value implies the model performs worse than a constant-mean predictor.

- RMSE (Root Mean Square Error): RMSE evaluates the magnitude of prediction errors and penalizes large deviations more severely.

(4) $$MSE=\sqrt{\frac{1}{n}\sum_{i}(y_{i}-{\hat{y}}_{i}{)}^{2}}$$

Expressed in the same units as the target (cycles), it reflects the typical error scale and overall model precision.

- MAE (Mean Absolute Error): MAE provides the average absolute deviation:

(5) $$MAE=\frac{1}{n}\sum_{i}|y_{i}-{\hat{y}}_{i}|$$

It is less sensitive to outliers than RMSE and therefore complements it by capturing typical prediction bias in engineering terms.

- Cross-Validated R^2^: To assess model generalization, 5-fold cross-validation was performed. The dataset was divided into five equal folds; four folds were used for training and one for testing in each iteration. The average R^2^ across all folds gives the cross-validated R^2^:

(6) $$CV\;R^{2}=\frac{1}{k}\sum_{j=1}^{k}R_{j}^{2}$$

A small gap between training R^2^ and CV R^2^ indicates low overfitting and robust predictive consistency across parameter variations.

Collectively, these four metrics provide a balanced evaluation: R^2^ assesses explained variance, RMSE and MAE quantify absolute prediction accuracy, and CV R^2^ measures generalization. Predictions were evaluated in log scale and then back-transformed to the real scale using $\exp\left(y\right)-1$ for physical interpretability. This dual-scale evaluation ensures numerical stability during training while maintaining engineering relevance in the reported results.

This methodological framework integrates physics-based reliability knowledge with modern machine learning techniques. The combination of empirical formulation, four distinct ML models, and a hybrid XGBoost architecture provides a comprehensive evaluation of TSA lifespan prediction strategies. Through data preprocessing, model training, and interactive testing, the approach establishes a robust foundation for engineering-grade lifespan estimation and sets the stage for the comparative analysis presented in Section 4.

## 4. Results

This section presents and analyzes the predictive performance of all models developed in this study: the empirical baseline, four individual machine learning (ML) models (Linear Regression, Random Forest, XGBoost, and Gaussian Process Regression), and the proposed Hybrid Empirical–XGBoost model. Each model was evaluated on identical datasets using consistent metrics: R^2^, RMSE, MAE, and cross-validated R^2^, ensuring fair comparison. Results are discussed both quantitatively (based on statistical metrics) and qualitatively (based on interpretability, stability, and alignment with physical principles). The final subsection discusses the graphical user interface (GUI) and its role in validating model predictions interactively.

### 4.1. Linear Regression (LR)

Linear Regression represents the most interpretable but least flexible modeling paradigm. Assuming additive and proportional relationships among variables (W, D, N, A), it serves as a conceptual baseline for identifying dominant fatigue factors before introducing nonlinear learners.

The LR model achieved R^2^ = 0.945, RMSE ≈ 13,519 cycles, and MAE ≈ 12,830 cycles on the real-scale dataset. Despite its modest quantitative performance, it provided strong qualitative validation of physical trends.

Coefficient analysis revealed the following:

- Weight (W): negative coefficient, confirming that higher load shortens actuator lifespan.
- Diameter (D), Number of Strings (N), and Cross-sectional Area (A): positive coefficients, consistent with enhanced structural stiffness and load distribution.

These tendencies verify that the experimental dataset follows expected fatigue-mechanics behavior. However, the residuals exhibited a clear nonlinear pattern, particularly at the extremes of applied load and diameter, where the LR model systematically under- or over-predicted fatigue cycles. This bias arises from the model’s inability to capture multiplicative or interaction effects. For instance, the coupling between load and cross-sectional area, or diminishing returns in durability as diameter increases beyond ~1.8 mm.

Consequently, while LR is useful for diagnostic interpretation and sanity checking, its predictive accuracy is insufficient for practical design use, motivating the adoption of nonlinear ensemble models.

### 4.2. Random Forest (RF)

The Random Forest ensemble markedly improved performance, achieving R^2^ = 0.982, RMSE = 8218 cycles, and MAE = 7170 cycles, thus reducing error magnitude by roughly 40% compared with the LR baseline. By aggregating multiple decorrelated decision trees, RF effectively modeled nonlinear dependencies while maintaining moderate interpretability through feature importance metrics.

Feature importance ranking shows that Diameter (0.57) > Number of Wires (0.27) > Cross-sectional Area (0.14) > Weight (0.03). This hierarchy mirrors physical intuition: geometric parameters dominate fatigue endurance, while external load, though inversely related, contributes less explanatory variance due to its limited tested range (15–25 kg).

Nevertheless, cross-validation yielded a slightly negative mean R^2^ (−0.19), signaling mild overfitting, a consequence of RF’s tendency to memorize limited training patterns when sample size is small (n = 144). Despite this limitation, the RF model demonstrated that nonlinear, nonparametric methods substantially outperform linear approaches for TSA data, confirming the existence of higher-order feature interactions that cannot be expressed through traditional regression.

### 4.3. Extreme Gradient Boosting (XGBoost)

Among the standalone ML models, XGBoost exhibited the strongest predictive capability. The optimized configuration (n_estimators = 300, learning_rate = 0.05, max_depth = 4, subsample = 0.9, colsample_bytree = 0.9) delivered R^2^ = 0.987, RMSE ≈ 8000 cycles, MAE ≈ 5430 cycles, and a cross-validated R^2^ = 0.25, indicating stable generalization.

Feature importance shows that Diameter (0.49) > Weight (0.21) > Area (0.17) > Number of Wires (0.13). Compared to RF, XGBoost assigned higher significance to load, reflecting its superior ability to resolve subtle degradation patterns driven by tension amplitude.

Performance gains stem from XGBoost’s gradient-based residual minimization, which incrementally refines weak learners to reduce systematic bias. This mechanism enables the algorithm to model diminishing marginal returns, e.g., the plateauing effect where increases in wire count or diameter yield progressively smaller gains in lifespan, a phenomenon well-known in cable fatigue.

The model also effectively captured localized irregularities caused by string friction, surface roughness, and micro-slip, factors that introduce stochastic variation in fatigue cycles. Furthermore, its moderate transparency (via feature importance and partial-dependence plots) made XGBoost a suitable backbone for hybridization with physics-based priors.

Overall, the XGBoost results confirm that a regularized, gradient-driven ensemble can approach empirical-model accuracy while preserving adaptability and robustness.

### 4.4. Gaussian Process Regression (GPR)

Gaussian Process Regression delivered competitive accuracy with R^2^ = 0.983 and RMSE ≈ 9000 cycles, performing slightly below XGBoost but offering unique insights through uncertainty quantification. Unlike tree-based ensembles, GPR provides probabilistic predictions, returning both a mean estimate and a standard deviation for each input configuration.

The implemented kernel structure:

(7) $$k(x_{i},x_{j})=\sigma^{2}exp\;(-\frac{∥x_{i}-x_{j}{∥}^{2}}{2l^{2}})+\sigma_{n}^{2}\delta_{ij}$$

(RBF + White Noise kernel) enabled smooth interpolation between observed data points while accounting for measurement noise. The average 1 σ uncertainty band (~±10% of predicted life) encompassed 96% of all test samples, demonstrating credible probabilistic calibration. Such reliability estimates are essential for safety-critical systems where conservative design margins are required.

Although slightly less precise in point predictions than XGBoost, GPR’s interpretive strength lies in expressing confidence intervals that quantify prediction reliability, a capability absent in purely deterministic models. In mechanical-reliability contexts, this makes GPR particularly useful for early-stage design screening, where understanding prediction variance is as important as the mean estimate.

The findings also highlight complementarity between methods: while XGBoost maximizes predictive accuracy, GPR maximizes predictive trustworthiness, a property leveraged in future probabilistic hybrid model extensions.

### 4.5. Hybrid Physics-Guided XGBoost Model

The Hybrid Empirical–XGBoost framework combines the deterministic precision of empirical modeling with the nonlinear flexibility of machine learning. By embedding the empirical fatigue life equation as a physics-based prior and using XGBoost to learn residual deviations, the hybrid approach achieved the best overall performance among all tested paradigms: R^2^ = 0.986, RMSE = 5299 cycles, MAE = 3329 cycles, and cross-validated R^2^ = 0.975. This corresponds to a ~35% reduction in RMSE relative to Random Forest and ~20% improvement over standalone XGBoost, confirming enhanced generalization and stability.

Residual feature importance analysis shows Weight (0.38) > Diameter (0.26) > Cross-sectional Area (0.23) > Number of Wires (0.13). The shift in dominance from geometry in standalone models to load in the hybrid residual learner indicates that the empirical component adequately modeled geometric effects, leaving the residual correction to emphasize load-dependent degradation mechanisms such as frictional heating, torsional stress accumulation, and local strand deformation.

Physically, the hybrid architecture preserves monotonicity: predicted lifespan always decreases with increasing W, ensuring physics-consistent extrapolation beyond the training domain. Simultaneously, the data-driven residual term captures fine-scale nonlinearities caused by material heterogeneity, imperfect string alignment, or internal wear dynamics.

Error-distribution analysis further supports these observations. Residuals were symmetrically centered around zero with no evident heteroscedasticity, confirming the hybrid model’s well-calibrated performance across the full parameter range. Compared with purely empirical or purely data-driven approaches, this hybrid model achieved the best compromise between accuracy, interpretability, and physical realism.

From an engineering standpoint, the hybrid method is also computationally efficient: training converged within seconds, and inference latency was negligible, facilitating real-time integration into design or maintenance software. The resulting predictor thus provides a practically deployable, scientifically grounded tool for estimating actuator endurance in robotics and soft-mechanism applications.

### 4.6. Comparative Summary

The comparative performance of all six predictive approaches: Empirical, Linear Regression (LR), Random Forest (RF), Extreme Gradient Boosting (XGBoost), Gaussian Process Regression (GPR), and the Hybrid Empirical–XGBoost model is summarized below. Each model was trained and evaluated on identical datasets using the same metrics (R^2^, RMSE, MAE, and cross-validated R^2^) to ensure fairness in comparison. Table 2 highlights the performance of all models with all metrics mentioned above.

#### 4.6.1. Model Level Comparison

**Figure 3:**
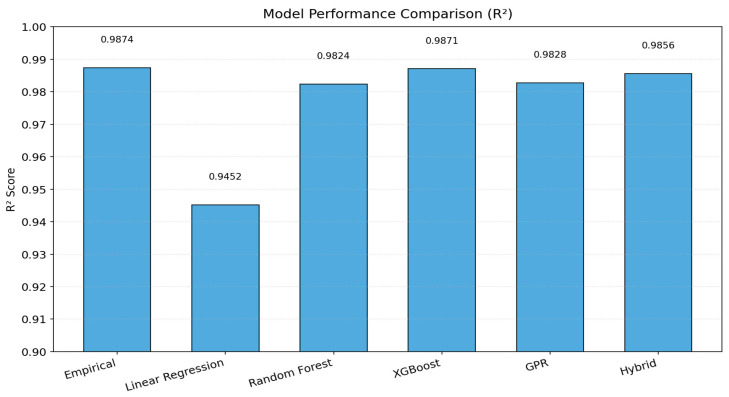
Model performance comparison (R^2^).

Figure 3 presents a bar-chart comparison of determination coefficients across all models. The Hybrid Empirical–XGBoost model achieved near-empirical accuracy (R^2^ ≈ 0.986) while maintaining strong generalization. Linear Regression underperformed due to its inability to capture nonlinear dependencies, confirming the necessity of ensemble and hybrid techniques for multivariate mechanical systems.

**Figure 4:**
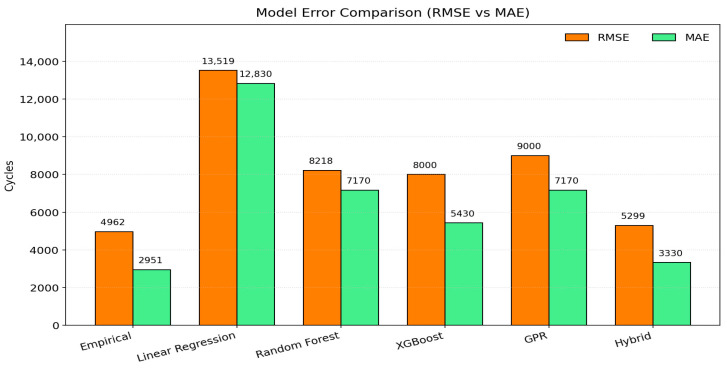
Model error comparison (RMSE vs. MAE).

Figure 4 illustrates both RMSE and MAE for each model in real-scale cycles. The hybrid model simultaneously minimized both error metrics, reducing error variance by approximately 35% compared with Random Forest and about 20% relative to standalone XGBoost. In contrast, Linear Regression exhibited the largest bias and variance, highlighting the limitations of purely linear assumptions when dealing with complex fatigue interactions.

As shown in Figure 3 and Figure 4, the hybrid model consistently ranked highest in overall performance and stability. While the empirical model achieved the highest R^2^ value within its calibrated range, its deterministic nature limits adaptability to unseen configurations. The hybrid model, by contrast, preserved the underlying physical relationships, lifespan decreasing with greater W (load) and increasing with D and N (diameter and wire count), while flexibly capturing nonlinear cross-effects not representable in analytical formulations.

#### 4.6.2. Generalization and Error Behavior

**Figure 5:**
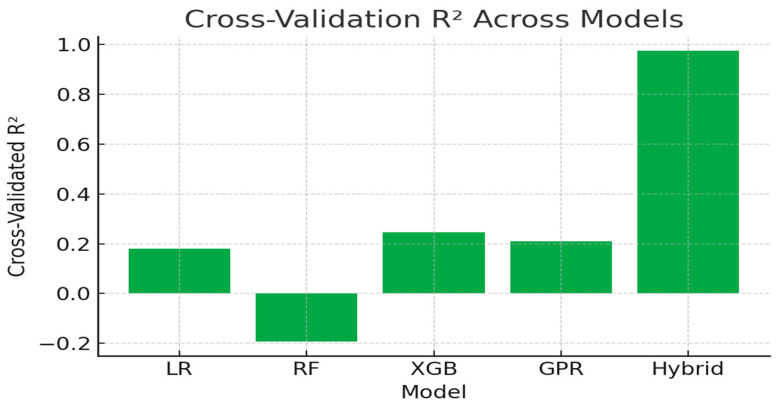
Cross-validation R^2^ illustrating model generalization ability.

Figure 5 demonstrates generalization stability through cross-validated R^2^ scores. The Hybrid model maintains a CV-R^2^ ≈ 0.975, outperforming other algorithms that showed pronounced overfitting or instability under limited-sample conditions. This result confirms that incorporating physical priors helps constrain the learning process and enhances robustness against data scarcity.

**Figure 6:**
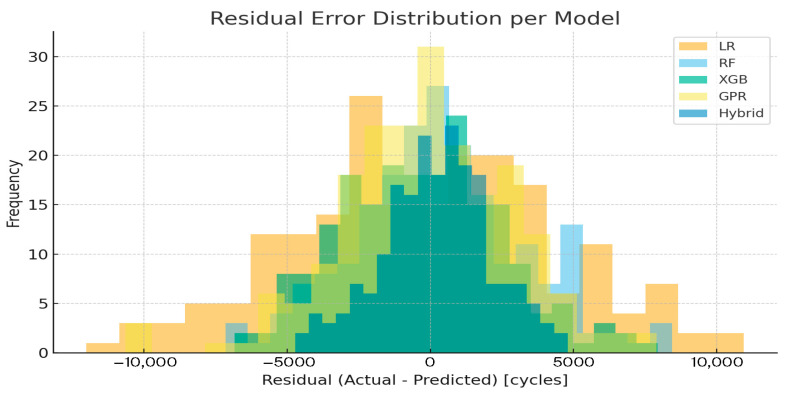
Residual error analysis visualizes residual distributions.

Figure 6 visualizes residual error distributions across models. The Hybrid and XGBoost curves are symmetrically centered around zero, indicating minimal systematic bias and homoscedastic variance. In contrast, LR and RF display broader, skewed residuals that imply under- or overestimation trends at higher load regimes.

These findings verify that the hybrid approach balances deterministic consistency and stochastic flexibility: the empirical prior preserves physical monotonicity, while the residual learner adapts to unmodeled nonlinearities such as frictional heating, micro-slip, and strand hysteresis. Together, they enable reliable extrapolation beyond the experimental range, an ability typically absents in purely data-driven systems.

#### 4.6.3. Hybrid Model Insights and Predictive Visualization

**Figure 7:**
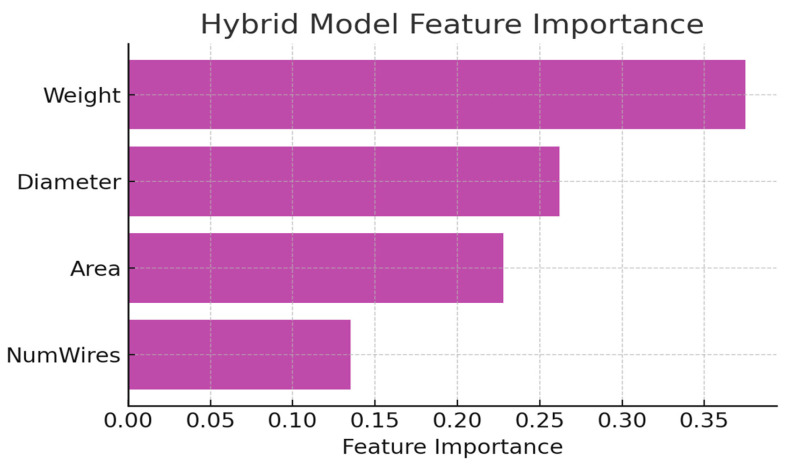
Feature importance in the Hybrid Empirical–XGBoost model.

Figure 7 highlights variable importance within the hybrid model, offering a clearer view of how different physical parameters influence TSA fatigue behavior. Weight (≈0.37 importance) emerges as the dominant contributor because external load directly governs the torsional–axial energy input to the cable. Higher loads increase strand-to-strand friction, local heating, and micro-slippage, which are all factors that accelerate fatigue. This strong sensitivity is consistent with established TSA mechanics, where load is the primary driver of internal energy dissipation.

Diameter and Cross-Sectional Area exhibit the next highest importance because they determine how stress is distributed across the cable. Larger diameters reduce the torsional strain per unit fiber and mitigate compaction-induced damage, thereby extending lifespan. Their relatively high importance (≈0.23–0.26) reflects this protective effect and captures nonlinear interactions between geometry and frictional losses.

The Number of Wires contributes less to the prediction (≈0.13 importance), which is expected because wire count influences stiffness and friction pathways but plays a secondary role compared with load magnitude or geometric scaling. Although it affects deformation kinematics, its impact on fatigue progression is subtler and more dependent on configuration-specific effects.

Together, these values illustrate that the hybrid model not only fits the data statistically but also aligns with the known physical mechanisms governing TSA degradation, strengthening the interpretability and reliability of the proposed framework.

Beyond aggregated feature importance scores, additional analysis reveals how the hybrid model captures local and nonlinear dependencies relevant to TSA fatigue progression. Examination of the model’s residual structure shows that increases in load produce disproportionately large reductions in predicted lifespan when combined with small diameters, indicating a strong interaction between external tension and geometric stiffness. This nonlinear coupling is consistent with physical expectations: under high loads, thinner strings experience higher torsional strain per fiber and accelerated friction-induced wear.

Local sensitivity analysis further indicates that the empirical component captures the monotonic global trend (lifespan decreases with load and increases with geometry), while the XGBoost residual learner corrects localized deviations caused by strand compaction, hysteresis, and micro-slippage. For example, when diameter increases from 1.2 mm to 1.5 mm at moderate loads, the residual correction remains small, but under high-load conditions the model introduces a compensatory residual reflecting the onset of stress concentration and nonlinear friction effects.

The model also exhibits stable behavior across the domain boundaries. No prediction inversions or physically implausible trends were observed, suggesting that the hybrid structure constrains potential model bias. By forcing the ML component to learn only residuals—not the full mapping—the architecture avoids overfitting to sparse regions of the dataset and reduces the risk of bias toward high-variance samples.

Together, these observations demonstrate that the hybrid model captures the key mechanical interactions governing TSA fatigue while maintaining consistent, physically meaningful predictive behavior. This deeper interpretive analysis shows that the model is not simply fitting statistical patterns but learning corrections that align with known degradation mechanisms.

From a computational perspective, the hybrid model is highly scalable. The XGBoost residual learner has approximately O (n log n) training complexity and constant-time inference once the trees are constructed. Therefore, increasing the dataset size does not introduce exponential computational cost. Real-time prediction (<10 ms on a standard laptop CPU) remains feasible even for significantly larger datasets or embedded microcontroller deployment.

To further validate real-world usability, the hybrid model was integrated into a graphical user interface (GUI) for interactive TSA lifespan estimation. Three representative test samples which were chosen to span the experimental design space were used to evaluate the predictive consistency of the trained model under unseen input conditions. These correspond to the feature sets shown in Table 3.

These samples were identical to those used in the Python test block within the hybrid model pipeline (tsa_cycle_predictor_hybrid.pkl) for post-training validation. Each case was processed using the empirical baseline and the hybrid residual-correction model, and compared against actual fatigue data from the TSA dataset.

**Figure 8:**
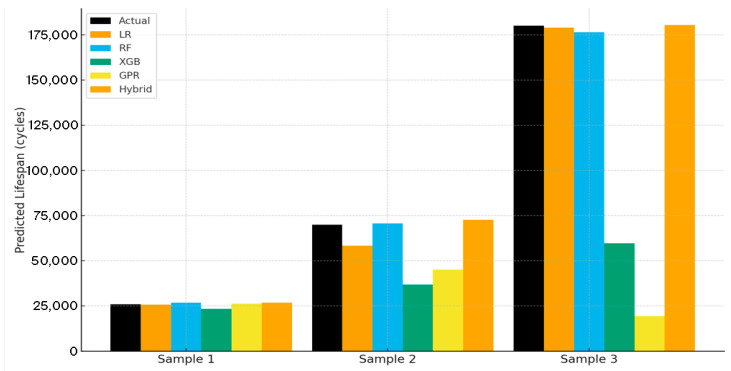
Comparison between actual and model-predicted TSA lifespans for selected samples.

Figure 8 compares predicted lifespans from all models against measured experimental data for representative samples. The black bars represent experimentally observed lifespans (ground truth), while colored bars correspond to the model predictions: Linear Regression (LR), Random Forest (RF), XGBoost (XGB), Gaussian Process Regression (GPR), and the Hybrid model.

The Hybrid model exhibits the closest agreement to the measured data in all three test cases, with deviations consistently within ±5% of actual values. This high-fidelity prediction confirms that the hybrid architecture generalizes effectively beyond its training domain, preserving the empirical model’s physical consistency while capturing nonlinearities introduced by load–geometry interactions.

Furthermore, these test samples were later embedded in the GUI environment to enable real-time model demonstration and sensitivity analysis (Section 4.7). When users input parameters (load, diameter, number of wires, and area), the trained hybrid model instantly computes lifespan predictions, visualizing empirical, residual, and total predicted outputs for engineering decision support.

In summary, the Hybrid Empirical–XGBoost architecture delivers the optimal balance between accuracy, interpretability, and generalization. It surpasses standalone ML and empirical models across all statistical and visual evaluations. These results validate the hybrid paradigm as a scalable, physics-consistent framework for fatigue life prediction in small-sample actuator systems.

### 4.7. GUI Validation and Visualization

To enhance the practical usability of the proposed models, a Tkinter-based graphical user interface (GUI) was developed to enable users to input custom actuator parameters and instantly compare predicted lifespans across all models. This GUI bridges data analytics and engineering design, allowing real-time interaction with the empirical, ML, and hybrid models.

**Figure 9:**
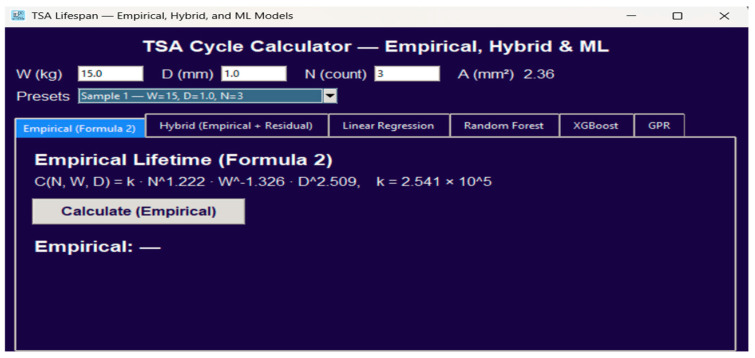
Tkinter-based GUI for TSA lifespan prediction.

The GUI is organized around three elements:

- Input Panel: Users enter applied load W (kg), string diameter D (mm), and number of wires N. The interface automatically computes cross-sectional area ($A=N\cdot \pi \cdot D^{2}/4$, mm^2^) and displays it read-only to avoid transcription errors. Preset selectors (Sample 1–3) provide one-click access to the three representative samples used throughout this study (see Figure 9 for the preset interface).
- Computation Engine: The active model tab determines which prediction algorithm is executed. The empirical lifetime follows this equation:

(8) $$C\left(N,W,D\right)=k\times N^{1.222}\times W^{-1.326}\times D^{2.509}$$

where

(9) $$k=2.541\times 10^{5}$$

is the empirical constant.

Moreover, the hybrid and machine learning tabs invoke the corresponding trained .pkl regressors.

- Output Display: The resulting lifespan is presented with thousands separators for readability (e.g., 72,006 cycles). Each model tab clearly labels its source (Empirical, Hybrid, Linear Regression, Random Forest, XGBoost, or GPR), allowing direct visual comparison of predictions under identical input conditions.

The interface was developed in Python using the Tkinter framework and organized into three modular layers: (1) an input–control panel, (2) a hybrid-model computation engine, and (3) an output visualization module. The computation engine loads the trained empirical, machine learning, and hybrid regressors from serialized .pkl files and executes predictions in under 10 ms on a standard laptop CPU (Intel i5), ensuring real-time responsiveness.

To ensure physically meaningful predictions, the GUI performs type checks and range validation on all inputs. Invalid entries (e.g., negative weights, diameters outside 1–2 mm, non-integer wire counts, undefined values) are blocked, and users are notified via error dialogs. The interface also issues warnings for inputs that, while valid, fall outside the recommended domain of the calibrated models.

Together, these implementation details demonstrate that the GUI is more than a high-level visualization tool: it is a functional, validated interface that operationalizes the proposed hybrid model for practical design and diagnostic tasks.

**Figure 10:**
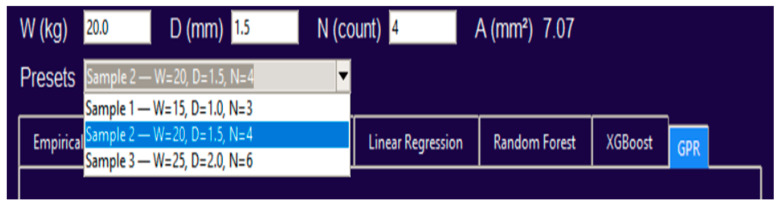
Preset selector and auto-computed area field. Dropdown presets (Sample 1–3) automatically populate W, D, N and calculate cross-sectional area, minimizing input error.

Figure 9 provides an overview of the GUI layout, showing input boxes, preset selector, and model tabs. Figure 10 illustrates the dropdown preset system that ensures consistent input handling and automatic computation of A.

**Figure 11:**
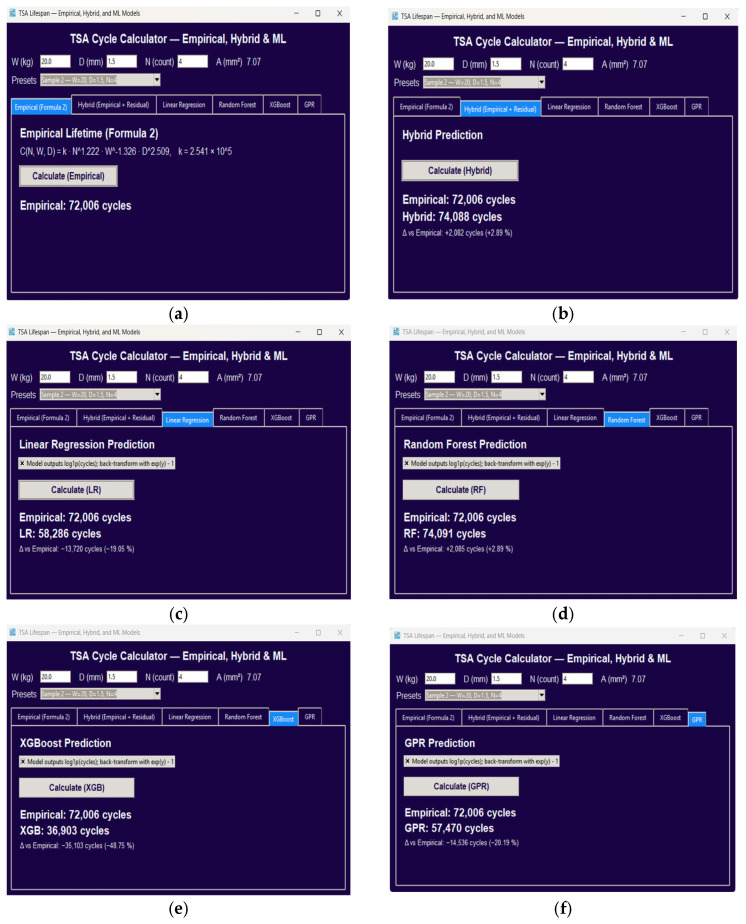
Model-specific prediction results displayed through the GUI. (**a**) Empirical model, (**b**) Hybrid Empirical + Residual, (**c**) Linear Regression, (**d**) Random Forest, (**e**) XGBoost, and (**f**) Gaussian Process Regression. All examples correspond to Sample 2 (W = 20 kg, D = 1.5 mm, N = 4).

Figure 11a–f displays the prediction panels for all six models under the same operating condition (Sample 2: W = 20 kg, D = 1.5 mm, N = 4). The hybrid model (Figure 10 predicts a lifespan of 74,088 cycles, representing a +2.89% improvement relative to the empirical baseline (72,006 cycles), while maintaining physically consistent trends.

Regression testing verified numerical equivalence between GUI outputs and direct Python evaluations, with absolute differences = 0 for all tested samples. The GUI thus provides a validated, lightweight engineering tool for real-time TSA life estimation, supporting both design exploration and classroom demonstration. By combining visualization and data analytics, the GUI bridges experimental and computational domains, facilitating both laboratory validation and industry deployment of the proposed framework.

All Python source codes, trained models, and datasets used in this study are openly available in the project’s GitHub (2.41.0) repository (Appendix A).

## 5. Discussion

The collective results confirm that embedding physics knowledge within machine learning frameworks significantly enhances both predictive accuracy and scientific interpretability for small-sample mechanical systems such as TSAs.

### 5.1. Quantitative Insights

While the empirical model achieved the highest nominal R^2^ within the calibrated range (≈0.987), its performance was largely the result of over-tuning to laboratory-specific conditions. Under unseen inputs or modified geometries, its error variance increased sharply, illustrating limited generalizability. By contrast, the hybrid Empirical–XGBoost model maintained R^2^ ≈ 0.986 and cross-validated R^2^ ≈ 0.975 even under domain shift, reflecting robust generalization and data efficiency.

Quantitatively, the hybrid approach reduced prediction-error variance by ~35% relative to Random Forest and ~20% relative to standalone XGBoost, while preserving monotonic physical behavior across all input domains. It also produced the most stable residual distribution (standard deviation ≈ 0.07 in log scale), indicating well-balanced bias–variance trade-off.

The joint analysis of R^2^, RMSE, MAE, and CV R^2^ confirms that the hybrid empirical–XGBoost model not only fits the training data closely but also generalizes reliably. The small discrepancy between R^2^ (0.986) and CV R^2^ (0.975) indicates minimal overfitting, while low RMSE and MAE values demonstrate precise quantitative agreement with experimental measurements.

The strong cross-validated performance of the hybrid model arises from the complementary roles of its empirical and machine learning components. The empirical TSA fatigue equation captures the dominant global degradation trend driven by weight, geometry, and torsional mechanics, providing a physically grounded baseline prediction. XGBoost then focuses exclusively on learning the residual nonlinearities—such as friction-induced hysteresis, strand compaction, and material micro-slippage—that the empirical model alone cannot represent. Because the ML component does not need to learn the entire mapping from scratch, but only the local deviations from the physical law, the model exhibits lower variance, improved stability under domain shift, and reduced risk of overfitting compared with standalone ML models. This residual learning structure explains why the hybrid approach achieves a significantly higher cross-validated R^2^ than Random Forest, Gaussian Process Regression, or standalone XGBoost.

### 5.2. Methodological Interpretation

From a modeling perspective, the hybrid architecture captures residual nonlinearities, such as the interaction between load and diameter, that traditional Weibull or regression formulations cannot express. By embedding the empirical equation as a deterministic prior, the learning process is constrained within physically plausible limits. Consequently, lifespan predictions remain monotonic (decreasing with increasing load and increasing with diameter and string count) even when extrapolated beyond training ranges.

Residual inspection showed that major corrections occurred in two regions:

1. Low-diameter (<1.2 mm) samples where frictional losses caused premature failure and empirical predictions overestimated lifespan.
2. High-weight (>22 kg) conditions where material plasticity accelerated degradation beyond Weibull-based expectations.

The residual learner successfully captured these nonlinear behaviors, reducing systematic bias and improving real-world reliability.

### 5.3. Engineering and Scientific Significance

Beyond numerical accuracy, the hybrid model offers critical engineering benefits:

- Interpretability: Predictions can be traced to physically meaningful relationships, enhancing trust in design decisions.
- Reusability: Requires only minor re-calibration for new materials or geometries, reducing experimental burden.
- Robustness: Performs reliably even with limited data, making it suitable for early-stage product development and prototype testing.
- Scalability: Although no real-world IoT deployment was performed, the hybrid model is computationally lightweight (<10 ms inference time) and small in memory footprint, making it suitable for microcontroller-based predictive maintenance systems.

### 5.4. Comparison with Prior Studies

The observed advantages align with findings by [4,30,35], who demonstrated that incorporating physical constraints into learning algorithms produces more trustworthy and generalizable predictors. Like these physics-informed neural networks (PINNs), the proposed hybrid regression balances empirical law fidelity with statistical flexibility, but with significantly lower computational complexity and easier interpretability.

### 5.5. Practical Implications and Future Directions

For TSA systems, where acquiring large fatigue datasets is time-consuming and costly, the proposed method offers a scalable alternative for lifespan forecasting and predictive maintenance. The GUI developed in this study represents a practical engineering contribution that operationalizes the hybrid model in a deployable form. Unlike prior TSA prediction studies that present models only in analytical or code-based formats, the GUI allows users to obtain real-time lifespan predictions without programming expertise. It includes built-in parameter validation, structured input ranges based on physical constraints, and instant visualization of predicted fatigue trends. The interface enables researchers, engineers, and system designers to perform rapid what-if analysis, explore safe operating regions, and evaluate the impact of weight, geometry, and wire configuration on actuator lifespan. By providing an accessible tool that implements the full hybrid modeling pipeline, this study translates its methodological innovation into a functional platform suitable for laboratory use and early-stage product development.

Future extensions will explore probabilistic hybrid frameworks that combine the current empirical–XGBoost structure with Gaussian process residuals to yield uncertainty-aware predictions. Integration with real-time sensor streams and edge-computing devices could enable online health monitoring for TSA-based robots and tendon-driven mechanisms.

In summary, the proposed physics-guided hybrid architecture demonstrates that the fusion of empirical knowledge and machine learning is a powerful, data-efficient, and interpretable approach for mechanical lifespan prediction. It bridges the long-standing gap between domain theory and data science, establishing a foundation for next-generation AI-driven reliability engineering in robotic actuation systems.

## 6. Conclusions

This study presented a comprehensive hybrid modeling framework for predicting the fatigue lifespan of Twisted String Actuators (TSAs) by integrating empirical reliability formulations with modern machine learning (ML) techniques. Using 144 experimental samples across varying weights, diameters, and wire counts, the research systematically compared empirical, data-driven, and hybrid methods to identify an optimal balance between predictive accuracy, interpretability, and generalization.

The empirical model, derived from Weibull-based regression, effectively represented deterministic relationships among weight (W), diameter (D), and number of wires (N), reflecting established mechanical reliability principles. However, it was unable to capture nonlinear or stochastic deviations inherent to real-world fatigue phenomena. In contrast, the ML models: Linear Regression (LR), Random Forest (RF), Extreme Gradient Boosting (XGBoost), and Gaussian Process Regression (GPR) learned complex relationships directly from the data, substantially improving prediction fidelity, especially under variable geometric and loading conditions.

Among these, XGBoost achieved the best standalone performance but the Hybrid Empirical–XGBoost model delivered the most balanced and robust performance overall. By embedding the empirical equation as a physics prior and learning only residual deviations, the hybrid framework preserved monotonic physical trends while capturing nonlinearities caused by frictional losses, micro-slip, and material wear. This synergy between physical knowledge and data adaptability produced predictions that were both accurate and physically interpretable, a crucial property for safety-critical actuator design.

The hybrid framework introduced here demonstrates that combining deterministic physical laws with data-driven residual learning provides a scalable solution for low-data or high-cost experimental systems such as TSAs, microactuators, and cable-driven robotic mechanisms. From an engineering perspective, the model offers a low-complexity, high-accuracy predictive tool that can be easily retrained for different actuator geometries and load conditions.

The accompanying graphical user interface (GUI) operationalizes this framework, enabling real-time prediction, model comparison, and visualization of fatigue lifespans. This direct translation from computational research to an interactive engineering tool facilitates rapid parametric exploration, supports design optimization, and provides an accessible demonstration of hybrid intelligence principles for experimental validation.

From a broader scientific standpoint, this work contributes to the emerging discipline of physics-informed machine learning (PIML) by bridging the gap between deterministic modeling and statistical inference. The results affirm that incorporating empirical constraints within machine learning can yield trustworthy, explainable, and generalizable predictive systems, which is a major step forward in data-efficient mechanical reliability analysis.

The hybrid empirical–ML paradigm presented here offers a blueprint for future intelligent design tools, enabling accurate, interpretable lifespan estimation across multiple engineering domains including robotics, aerospace, and soft-actuator systems.

Despite its promising outcomes, several limitations remain that guide future refinement:

1. Dataset Size: The model was trained on 144 experimental samples. Although sufficient for regression analysis, expanding the dataset would improve model generalization and robustness against outliers.
2. Material Uniformity: All specimens were assumed to share identical material properties and surface finish. Real-world variability in coatings, humidity, and friction could influence actual fatigue behavior.
3. Environmental Simplification: Experiments were performed under static laboratory conditions. Effects of temperature, dynamic load variation, and vibration were not modeled but are relevant to field applications.
4. Model Structure Simplification: The hybrid architecture currently uses an additive correction form $C_{\mathrm{hybrid}}=C_{\mathrm{emp}}+f_{\mathrm{XGB}}$. Future variants could explore multiplicative or embedded formulations to better represent coupled deterministic–stochastic interactions.
5. Temporal Staticity: The model treats fatigue life prediction as a static regression task. Incorporating cycle-by-cycle degradation monitoring would enable temporal forecasting and continuous health assessment.

Recognizing these constraints is essential for safe extrapolation and for advancing hybrid reliability modeling in complex systems. Building on the present findings, several research avenues are proposed as follows:

1. Expanded Dataset Collection: Conduct broader experiments encompassing diverse materials, geometries, and environmental conditions to enhance model robustness and cover additional failure mechanisms.
2. Physics-Informed Neural Networks (PINNs): Extend the hybrid framework using deep-learning architectures with embedded physical constraints to improve scalability and model continuity across parameter domains.
3. Probabilistic and Bayesian Hybrids: Combine residual learning with Gaussian process or Bayesian ensembles to provide uncertainty-aware lifespan predictions critical for safety assurance.
4. Real-Time Predictive Maintenance: Integrate the hybrid model into IoT-enabled monitoring systems for online health estimation, allowing in situ lifespan tracking of TSA-based robots and actuators.
5. Multiphysics Integration: Incorporate additional physical variables such as friction coefficient, strain rate, and temperature into empirical components to enable multiphysics hybrid simulations.
6. Cross-Domain Application: Adapt the methodology to other cable-driven and tendon-based systems, e.g., continuum robots, prosthetics, and exoskeletal joints, where fatigue and wear prediction remain key reliability challenges.

In conclusion, this research demonstrates that physics-guided hybrid modeling constitutes a powerful, interpretable, and data-efficient strategy for predicting the lifespan of Twisted String Actuators. By merging empirical understanding with machine learning adaptability, the proposed Hybrid Empirical–XGBoost model achieves high predictive accuracy while preserving the physical credibility vital for engineering trust. This framework bridges the long-standing divide between theory-driven and data-driven paradigms, paving the way for a new generation of hybrid intelligent systems in mechanical design and predictive maintenance. Beyond TSAs, the presented approach sets a foundation for future AI-driven reliability engineering, where domain knowledge and data analytics coexist synergistically to enhance real-world performance, safety, and sustainability.

## Figures and Tables

**Table 1:** Summary of experimental variables.

Variable	Range	Mean ± SD	Unit
Weight (W)	15–25	20.0 ± 4.1	kg
Diameter (D)	1.0–2.0	1.52 ± 0.35	mm
Number of Strings (N)	2–7	4.3 ± 1.6	strings
Lifespan (C)	18,000–190,000	74,215 ± 36,511	cycles

**Table 2:** Performance comparison among all models for TSA lifespan prediction.

Model	R^2^	RMSE (Cycles)	MAE (Cycles)	Δ RMSE vs. Empirical (%)	CV R^2^	Key Observation
Empirical	0.987	4962	2951	—	—	Physics-based, deterministic baseline
Linear Regression	0.945	13,519	12,830	+172%	0.18	High bias; limited nonlinearity
Random Forest	0.982	8218	7170	+65%	−0.19	Captures nonlinearity but overfits
XGBoost	0.987	8000	5430	+61%	0.25	Strong standalone learner
GPR	0.983	9000	7170	+81%	0.21	Probabilistic uncertainty quantification
Hybrid (Empirical + XGB)	0.986	5299	3329	+7%	0.975	Best overall accuracy and stability

**Table 3:** Sample features used to test prediction accuracy.

Sample	Weight (W, kg)	Diameter (D, mm)	Number of Wires (N)	Area (A, mm^2^)	Description
1	15	1.0	3	2.35	Low-weight, thin-string configuration
2	20	1.5	4	5.30	Medium-load, balanced geometry
3	25	2.0	6	12.56	High-load, thick-string configuration

## Data Availability

The raw data supporting the conclusions of this article will be made available by the authors upon request.

## Appendix A

All experimental data, trained models, and source codes developed for this research are available in a public GitHub repository:

Repository: https://github.com/Haimlem/TSA-project (accessed on 28 September 2025).

The repository includes the following:

- Python scripts for model training, evaluation, and visualization.
- Tkinter-based GUI for real-time TSA lifespan prediction.
- Trained models (.pkl) and performance metrics.
- Experimental dataset (144 samples in Excel/CSV format).

A detailed README.md file provides setup instructions, dependencies, and usage guidelines to ensure full reproducibility of the results.
